# Effect of calcination temperature on the properties and applications of bio extract mediated titania nano particles

**DOI:** 10.1038/s41598-021-80997-z

**Published:** 2021-01-18

**Authors:** N. Saikumari, S. Monish Dev, S. Avinaash Dev

**Affiliations:** 1 Department of Chemistry, RMK College of Engineering and Technology, Chennai, 601206 India; 2 Anna University, Chennai, 600025 India; 3 RMK College of Engineering and Technology, ME, Chennai, 601206 India

**Keywords:** Environmental sciences, Chemistry, Energy science and technology, Engineering, Materials science, Nanoscience and technology

## Abstract

In order to deal with the arising environmental issues across the globe at present nano particles with unique properties laid a benchmark in the name of nano catalysis. In this work the significance of calcination temperature on the thermal, electronic, structural and surface properties of a nano catalyst produced by sol–gel method using ultrasonic radiation against the disposal of toxic textile pollutants is studied in detail. The extract of tea leaves has been used as a bio-template during the synthesis to revise the crystallite size, surface area, optical absorption potential, and rate of agglomeration of nano sized grains by regulating their physico-chemical and surface properties. The influence of calcination in the transformation of single phased anatase titania to mixed phase anatase–rutile titania and the corresponding outcome in its photocatalytic activity employed in water treatment applications have been verified. The nano catalyst obtained is characterized by X-ray diffraction (XRD), Scanning electron microscopy (SEM), Transition electron microscopy (TEM), Fourier transform infrared spectroscopy (FT-IR), Thermo gravimetric analysis (TGA), Brunaueur Emmett Teller (BET) analysis, UV–Vis diffused reflectance spectroscopy (DRS-UV–Vis) etc. The mesoporosity of the particle was examined using Barrett Joyner Halenda (BJH) model. The enhanced photo catalytic efficiency (about 97.7%) of templated nano titania due to calcination is verified against Congo red, a textile dye under optimized conditions. The nano catalyst produced can be easily separated, recycled to support its economic feasibility.

## Introduction

Recent advanced techniques in the nano science and nano technology resulted in the generation of tuned nano materials and have explored their applications in new fields like catalysis, electronics, pigments, gas sensing devices, medicine^1^, water purification and energy. Unquestionably, the progression of appealing nanoparticles with tuned properties is of unbelievable importance for both academic network and production^2^. Material Science evolved itself as a research hotspot in the field of nanotechnology and it is about the synthesis, characterization and study of materials in the nanometer region^3^.

For the past two to three decades advanced oxidation processes of heterogeneous catalysis under water treatment techniques demanded catalysts with enhanced size dependent properties particularly in the case of semiconductor metal oxides and sulphides^4,5^. Photocatalysis process among AOPs mainly employed in the field of effluent treatment from industries with the help of hydroxyl radicals being produced during the procedure^6^. From fundamental and practical perspective, TiO_2_ has become one of the most wanted nano material^7–10^ due to its photo and chemical stability, tunable electronic, optical, surface and structural properties, being easily available, reusable and less toxic. But, large scale applications of titania has always been limited due to its inefficient absorption of Sun light owing to its wider band gap energy. The advantages and disadvantages of homogeneous as well as heterogeneous catalysis paves a basic necessity of a new system, which ought to be vibrant as homogeneous catalysis, also efficiently regainable as heterogeneous catalysis^11^. Studies have been focused to overcome this limitation by tuning their crystallite, surface and optical characteristics via novel alternatives like making use of chemical and bio material templates, metal/non-metal doping and blending of different light absorbing materials^12^. Doping of metals like Ag, Ni, Co, Au, Cu, V, Fe, Mg^13^ were already reported by scientists on tailoring the electronic, surface, thermal and crystalline nature and non-metals like C^14^, N^15^, F^16^, S^17^ for causing red shift thereby enabling visible light active photo catalytic degradation of organic pollutants.

Being eco-friendly, easily available, inexpensive, thermally and mechanically stable^11^, biomolecules like PEG^18^, lotus root^19^, pollen grain^20^, avocado^21^, gelatin^22^, starch^23^, rice straw^24^ etc. have been exploited by various scientists as templates and found to be highly promising as they are capable of bringing out hydrogen bonding interactions that could control the rate of agglomeration and effect uniform dispersion^25^. In addition, to diminish the creation of harmful byproducts, green synthesis of the nano particles using bio materials were developed^26^. It can positively impact the nanomaterials thus produced either by removing or limiting the generation as well as usage of toxic substances^27^.

In this work extract of tea leaf has been used as a template and is found to consist of functional groups of proteins, amino acids, carbohydrates, vitamins C & E and lipids^28^. These components proved to be capable of effecting various physico-chemical and surface characteristics of titania during the synthesis and hence to control the growth of titania particles^29^.

The photo activity of synthesized titania nano materials have been manipulated by the calcination at different temperatures and the calcined titania samples were examined against Congo red a textile dye^30^. Congo red in water bodies is highly undesirable being carcinogenic, and should be eliminated. In this study the visible light photo catalytic ability of nano titania samples was expected to be very remarkable by simultaneous influence of bio templates and calcination in causing bathochromic shift (red shift) of absorption of light. This may result in successful, highly profitable and novel approach to harness solar energy for scientific projects like visible light bioremediation of waste water. This principle is projected to be more preferable in hot countries like India. The prominent impact of calcination in increasing crystallite size, narrowing band gap energy, eliminating irregularities and strain^31^ was noted and verified.

## Experimental details

### Materials

AR Titanium Tetra Iso Propoxide (TTIP, 99%), iso propanol (98%), acetic acid and demineralized water used in the synthesis of titania particles were purchased from Sigma Aldrich. Tea leaf extract (TLE) was obtained from India Mart in the form of powder. Congo red dye was obtained from Sigma Aldrich. HCl and NaOH were of Fischer Scientific make.

### Preperation of a catalyst

About 0.01 mol of TTIP was mixed with 0.25 mol of iso propanol and sonicated for 15 min to ensure complete dissolution. To the clear solution obtained about 0.05 mol acetic acid and 1 g of leaf extract were added and sonication was continued for 30 more minutes to obtain a clear sol. The resulting sol was kept static for 12 h to get a gel. The gel was dried in oven at 110 °C overnight and calcined at different temperatures 400 °C, 600 °C and 800 °C for 5 h and named as NT1, NT2 and NT3. Nano titania sample without calcination is designated as NT.

### Characterization of a catalyst

In order to determine the crystallize size and phase purity of the synthesized catalyst samples, XRD diffractogram was obtained using an X-ray diffractometer which recorded radiation in the range of 2θ from 20° to 80° at a scan rate of 2° min^−1^ using Cu Kα (λ = 1.546A°) radiations at room temperature. Study of FT-IR was performed with Perkin-Elmer using KBr pellet technique where the samples were exposed to scanning between 4000 and 400 cm^−1^. UV–visible diffused reflectance spectroscopy analysis using Schimadzu with BaSO_4_ as a reference was implemented on the synthesized catalysts. The energy of the band gap was determined using the formula Eg = hc/λ and measured using Tauc equation,

$$\left( {\alpha {\text{h}}\upsilon } \right)^{{\text{n}}} = {\text{ const }}\left( {{\text{h}}\upsilon \, {-}{\text{ Eg}}} \right)$$

where α is the absorption coefficient, hυ is energy of the photon, and n is the electronic transition type, usually n = 2 for directly allowed transitions. The surface area and pore size distribution were examined by BET and BJH analysis using Quadrasorb surface analyzer with respect to nitrogen adsorption–desorption isotherms. So as to analyze the morphology and elemental composition of the synthesized catalysts, Quanta 200 ESEM electron microscope equipped with energy dispersive micro analysis was employed. The thermal stability, phase transition and other chemical phenomenon during the thermal decomposition of the synthesized catalyst sample was determined using SDT Q 600 V8. Aurora TOC analyzer was used to estimate the degree of mineralization of Congo red from the total organic carbon (TOC) content.

## Results and discussions

### TGA analysis

**Figure 1:**
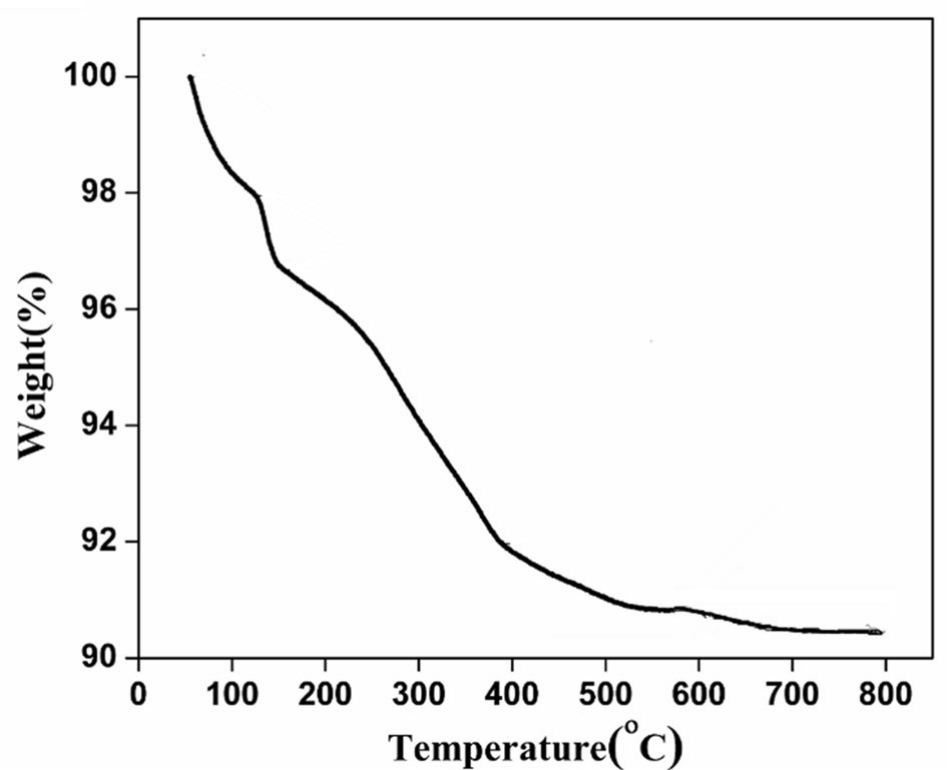
Thermal curve of as synthesized NT.

The thermal curve of as-synthesized nano titania sample is shown in Fig. 1. The initial stage of weight loss around 2% from 90 to 125 °C was due to the evaporation of moisture and other volatile compounds adsorbed on the surface^19^. The next stage of weight loss from 140 to 310 °C about 6.5% was due to the decomposition of poly phenols, carboxylic acid, poly saccharide, amino acid and other organic residues in the leaf extract^32^. The continuous weight loss of 1.2% from 325 to 450 °C, due to the removal of a template and other organic residues ensured the third stage of degradation^19^. The negligible change in weight from 400 °C indicated that the template has started to decompose and the process continued up to 800 °C. Hence the synthesized sample has been subjected to calcination at 400, 600 and 800 °C and the various interesting inferences have been noted. Since there is no considerable change in the weight after 400 °C, it is considered as optimum temperature for calcination.

### X-ray diffraction (XRD) analysis

**Figure 2:**
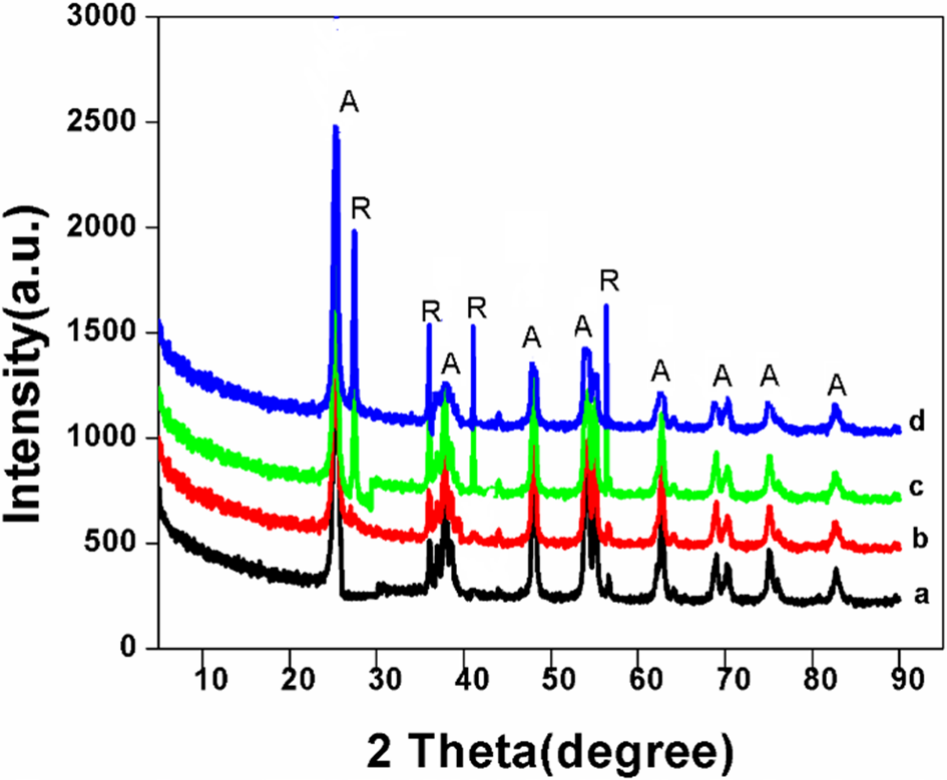
XRD patterns of (**a**) NT (**b**) NT1 (**c**) NT2, (**d**) NT3.

From the XRD patterns of the synthesized samples as shown in Fig. 2, the samples NT, NT1 exhibited the peaks at 2θ = 25.50 (101), 37.86 (004), 48.21 (200), 54.01 (105), 55.45 (211), 63.25 (204), 69.2 (116) and 75.2 (301) were in good agreement with the standard pattern (JCPDS 21-1272) for lattice planes of anatase titania. The non-existence of peaks corresponding to 27.5° and 30.8° have shown the absence of rutile and brookite phases^29^. Recording of noise level in the XRD diffractogram might be due to template particles on catalyst’s surface^12^.

Whereas in the case of NT2, NT3 samples, a weak diffraction peaks of rutile phase corresponding to (110), (101), (111) and (220) planes appeared at 27.6°, 36.2°, 41.6° and 56.4°, respectively. It is observed that, with the increase in calcination temperature the formation of thermodynamically stable rutile phase is favored, and has taken place gradually not abruptly. It is supposed to be due to the transformation of elongated side-to-side packed anatase phase to closed packed octahedraly oriented rutile phase^33^. Also it is noted that as the calcination temperature increased, due to phase transformation the intensities of the peaks corresponding to anatase phase decreased and vice versa. From Table 1, it is noted that with the increase in calcination temperature crystallite size is also increased. This is because at higher calcination temperature the formed crystallites are of larger size, which is attributed by the removal of irregularities, edges, defects etc. during heat treatment and therefore resulting in well-defined crystalline phase^34,35^. The average size of the synthesized nano titania samples NT1,NT2 and NT3 calcined at 400 °C, 600 °C and 800 °C were reported to be 18, 21.4 and 28.6 nm calculated using Debye–Scherrer formula (D = kλ/β cos θ)^36^ and was correlating well with the size determined from TEM analysis^37^.

**Table 1:** Physico-chemical parameters of the synthesized samples.

S. no.	Material	Crystalline size (nm)^a^	BET surface area (m^2^ g^−1^)^b^	Average pore radius (Å)^c^	Pore volume (cm^3^ g^−1^)^d^	Initial rate of Congo red degradation (mg l^−1^)
1	Degussa P25	50	50	–	–	0.05
2	NT	40.8	62.5	12	0.25	0.26
3	NT1	18	98.6	25.4	0.238	0.38
4	NT2	20.1	95.4	12.6	0.34	0.35
5	NT3	28.8	70.4	11.83	0.29	0.30

^a^Average crystalline size was determined by XRD using Scherrer equation.

^b^The BET surface area was determined from the linear part of the graph.

^c^Calculated from the desorption branches of isotherms.

^d^Estimated by BJH method.

**Figure 3:**
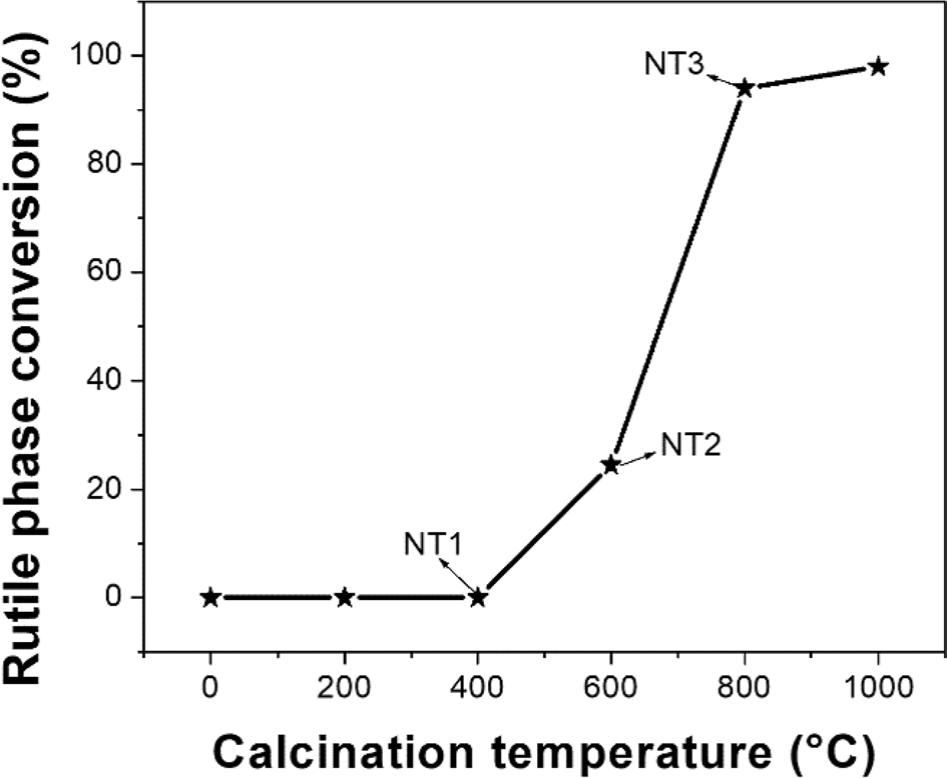
A–R transition during Calcination.

The percentage of rutile phase in NT2 and NT3 samples were determined using Spurr equation as, A (%) = 100/{1 + 1.265(IR/IA)} (Anatase %age), hence R (%) = 100 − A (%). It is found that A–R phase transition has occurred after 400 °C and continued even after 800 °C^26,38^. The recorded values of rutile phase percentage were 0, 25.8 and 94.6%, respectively for NT1, NT2 and NT3 (Fig. 3), thus A–R phase transition is almost near the completion at 800 °C.

### Fourier transform-infrared (FT-IR) analysis

**Figure 4:**
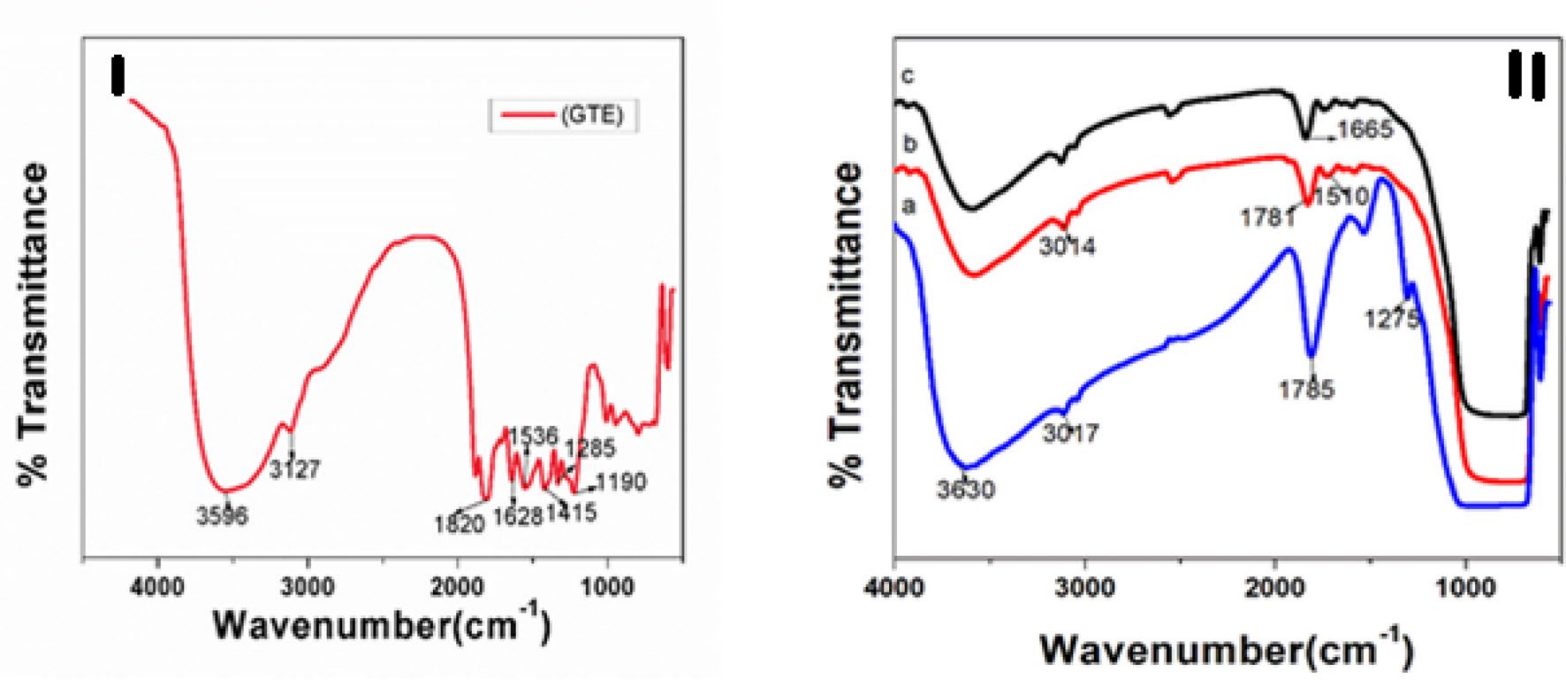
FT-IR spectrum of (**I**) tea extract, (**II**)a As-synthesized NT1, (**II**)b Calcined NT1, (**II**)c Calcined NT2.

Figure 4 gives the detailed account on FT-IR analysis of raw extract and templated titania samples. Changing di pole moments of molecular bonds lead to vibrational effects in the IR region^39^. From Fig. 4I, the FTIR spectrum of tea leaf extract has shown the peaks at 1624, 1396, 1299 and 1027 cm^−1^ corresponding to C=C, C–N, C–O–C and C–O stretching vibrations depicting the presence of poly phenols, carboxylic acid, poly saccharide and amino acid in the leaf extract^40^. The peaks at 3470, 2926, 2864 and 1752 cm^−1^ is attributed to O–H, asymmetric CH_2_, symmetric CH_2_ and C=O stretching vibrations respectively^41^. With respect to Fig. 4IIa, in as synthesized NT1, the shifting of peaks to the lower wave number indicated that the different functional groups of a template has involved in weak interaction with the metal Ti and oxygen on the surface of titania. The absence of characteristic peaks of a leaf extract template in the calcined NT1 and NT2 samples as shown in Fig. 4IIb,c, clearly portrayed the removal of an organic template during the heat treatment at 600 and 800 °C. The absorption band appeared around 400–800 cm^−1^ confirmed the formation of Ti–O–Ti linkage^42^.

### DRS-UV–visible spectra analysis

**Figure 5:**
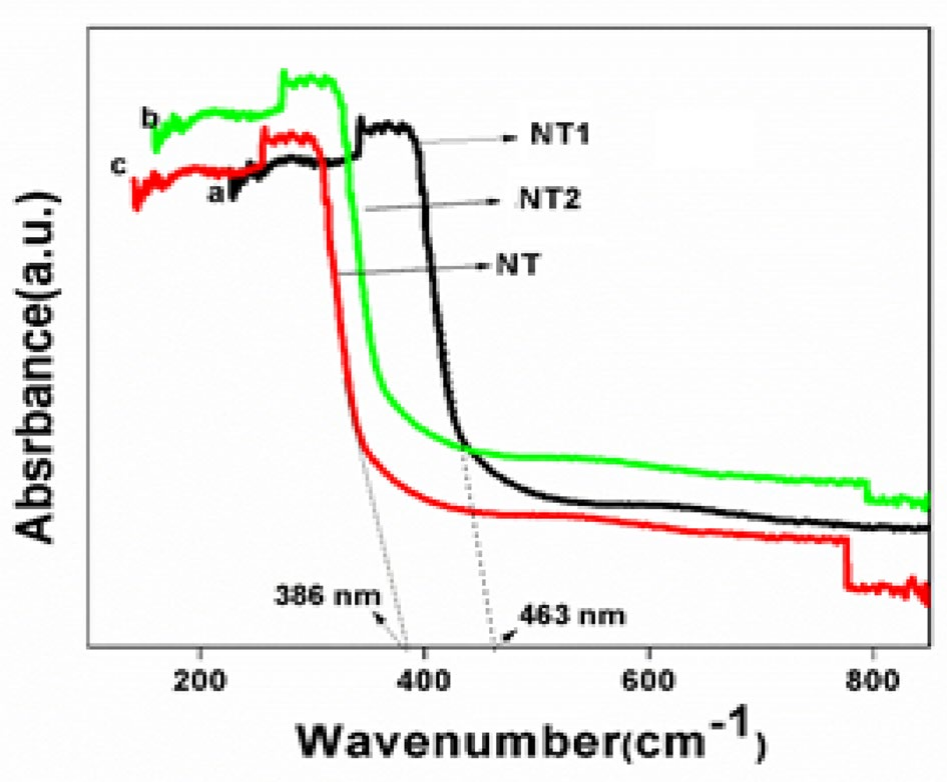
UV–Vis spectra of templated samples.

**Figure 6:**
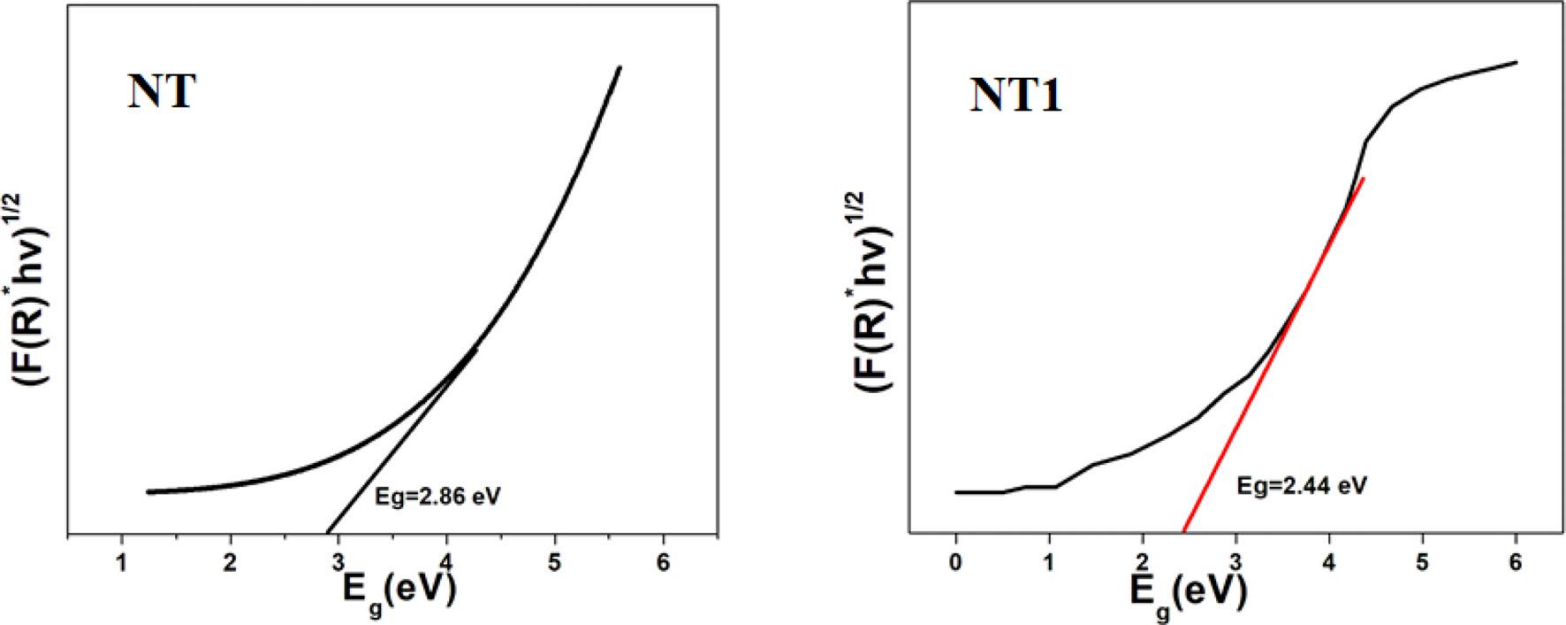
Tauc plot of NT and NT1.

Figure 5 shows the diffuse reflectance spectrum of templated titania samples. The absorption edge of a synthesized catalysts has shown a remarkable shift from UV to visible region. In the case of NT it appeared at 386 nm and for NT1 at 463 nm. This bathochromic shift (red shift), i.e. shifting of absorption edge towards longer wavelength, might be due to the delocalization of molecular orbitals in the least unoccupied conduction band of semiconductor metal oxides^43^. The above result is sustained from the band gap energy data observed from the Tauc plot (Fig. 6). The band gap energy of NT and NT1 samples were found to be at 2.86 and 2.44 eV, respectively, therefore in the case of NT1, the rate of recombination of excitons got lowered and thus increased the rate of photocatalysis overall. The above results inferred that NT1 could work as a visible light active photo catalyst in the degradation of organic pollutants and also it showed better results among the catalysts synthesized as shown in the analysis of photo catalytic efficiency analysis (Fig. 11).

### Surface characterization

#### Surface area (BET) analysis

**Figure 7:**
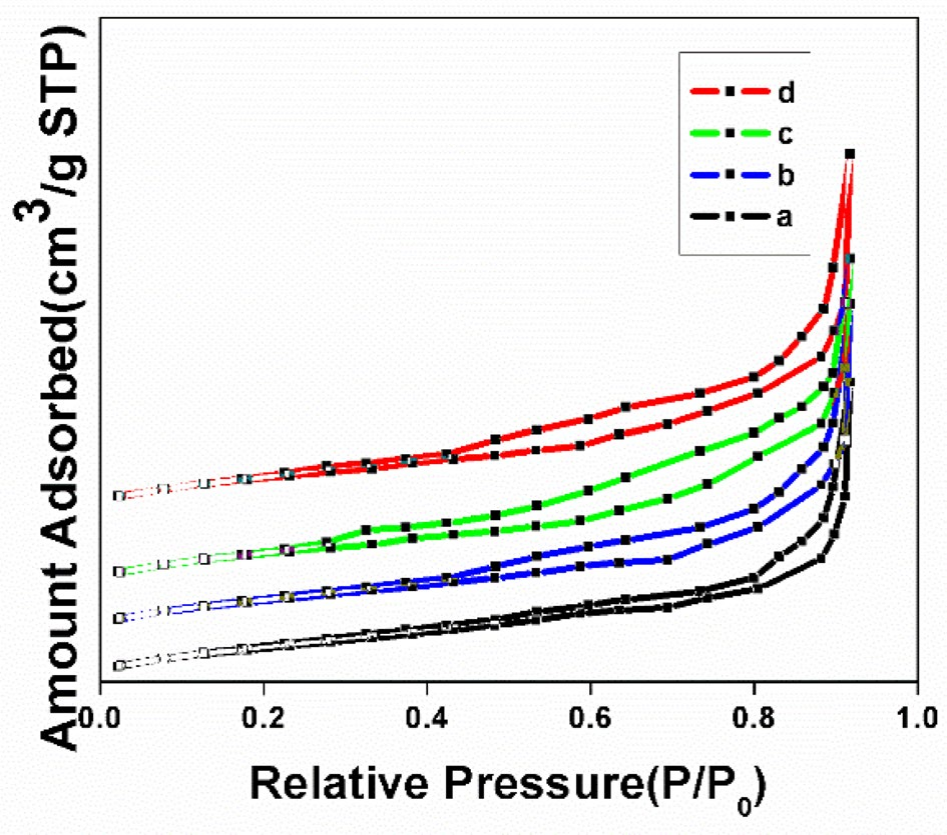
Nitrogen adsorption–desorption isotherm of (**a**) NT, (**b**) NT1, (**c**) NT2 and (**d**) NT3.

**Figure 8:**
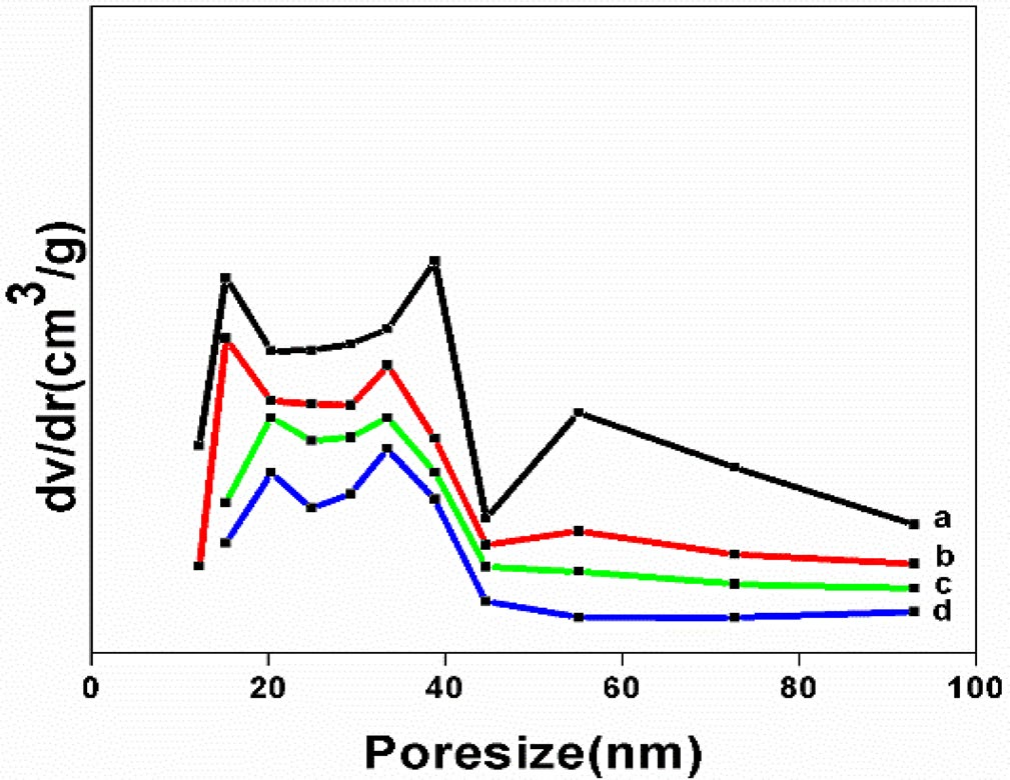
Pore size distribution curves of (**a**) NT1 (**b**) NT2 (**c**) NT3 and (**d**) NT.

Figure 7 shows the nitrogen adsorption desorption isotherm of NT, NT1, NT2 and NT3. The pore size, pore volume and the surface area of the resulting catalysts found to increase with the addition of leaf extract during the synthesis process and the values are shown in Table 1. From BET analysis it is inferred that all the synthesized samples exhibited the Type III adsorption isotherms^44^, with typical H3 hysteresis loop due to capillary condensation on the surface of mesoporous materials^45^. The enhanced mesoporosity of the templated nano titania samples compared to NT was validated from the broadening of the desorption portion of the isotherms of the former. Increase in surface area of about 30–65% was reported in templated samples in comparison with NT, among which NT1 showed the maximum surface area of 98.6 m^2^ g^−1^. This was in good agreement with the fact that lower the crystallite size greater will be the surface area of the metal oxide catalysts^46^. The deviation from the above fact in the case of other titania samples might be due to higher rate of agglomeration and lack of irregularities and defects on the catalyst’s surface because of calcination^47^. Moreover, the crystallite size derived from BET examination using the expression, D BET = 6000/S BET × D XRD nm, was found to be in well concurrence with the same that is assessed from the XRD pattern^48^. From the pore size distribution it was clear that the pores of the catalysts (Fig. 8) were narrow, bi modal and in the range from 11.5 to 13.5 nm. To summarize the impact of leaf extract as an effective template in the synthesis of mesoporous titania along with heat treatment resulted in expanded desorption isotherm, moderate porosity and narrowed pore size of NT1, is quite evident from its photo catalytic activity^49^.

**Figure 9:**
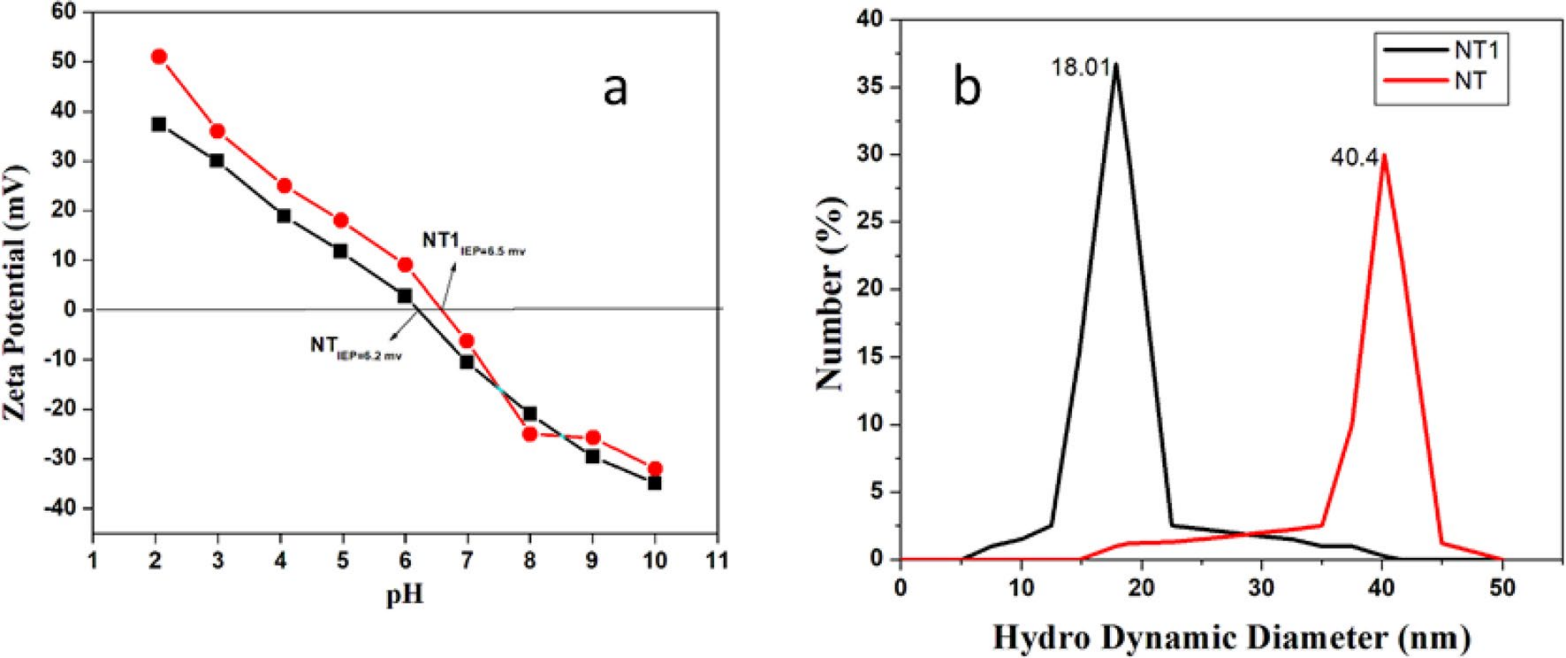
(**a**) Variation of Zeta Potential with pH (**b**) DLS pattern of NT and NT1 samples.

The zeta potential of NT and NT1 samples synthesized as a function of pH is shown in the Fig. 9a. The IEP of NT and NT1 was reported to be 6.2 and 6.5 mV, according to which the increase in the value might be caused due to fluidic stability of NT1 in aqueous suspension^50^. The DLS pattern with respect to Fig. 9b exhibits the particles size distribution of NT and NT1. The decrease in the particle size of NT1 compared to NT confirmed lowered agglomeration and also signified improved fluidic stability^50^.

#### Morphology and elemental (SEM and EDAX) analysis

**Figure 10:**
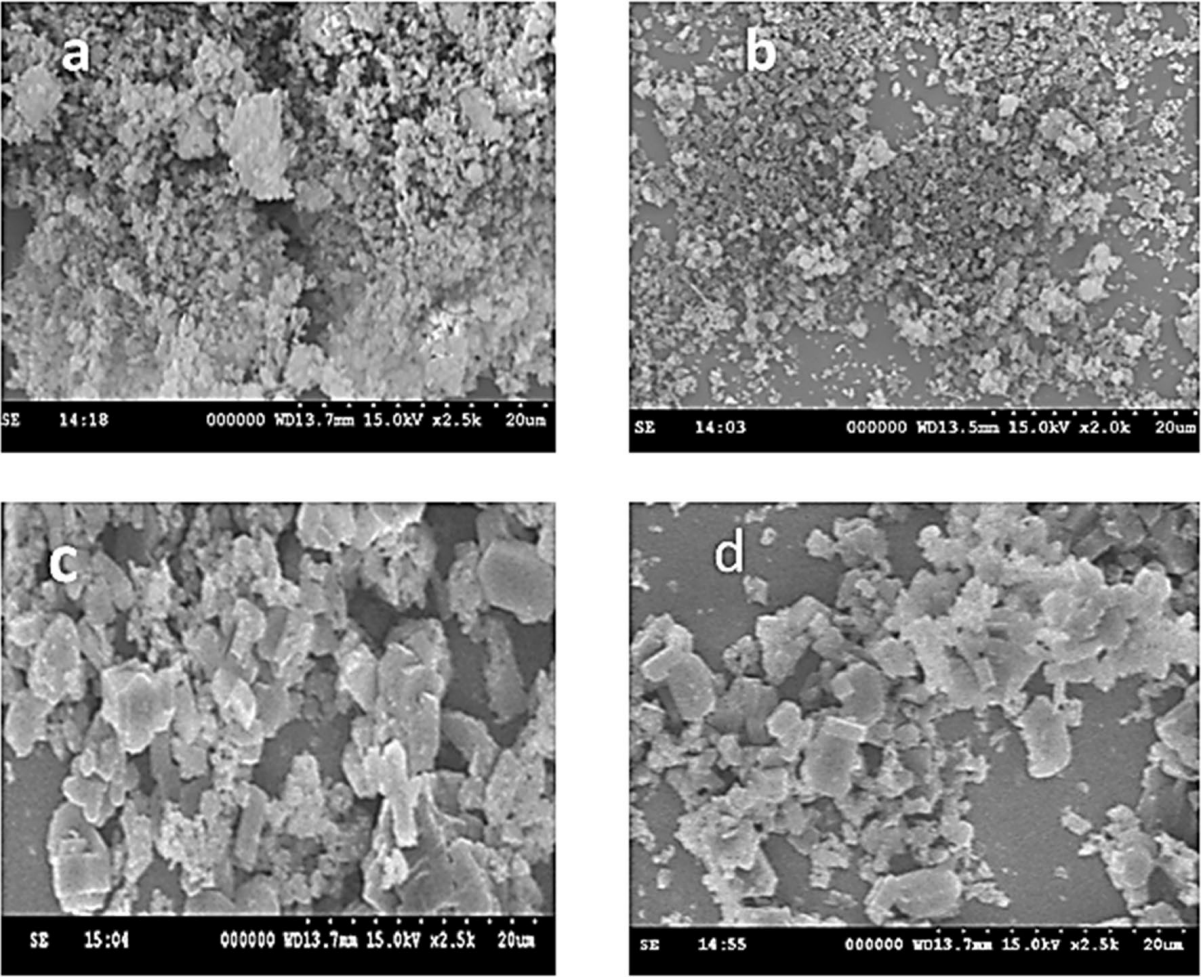
SEM images of synthesized (**a**) NT, (**b**) NT1, (**c**) NT2 and (**d**) NT3.

From the SEM analysis as shown in Fig. 10, the influence of a template on the morphology of titania nano particles has been verified. Among the templated titania samples NT1 showed moderate porosity, lowered agglomeration and well-dispersed nano clusters with inter crystalline pores, hence better morphology in comparison to other synthesized samples got reflected in its photocatalytic activity. Thus it is evident that lowered agglomeration reduce the crystallite size of the catalyst as observed in the BET surface area analysis. NT2 and NT3 samples have shown higher rate of agglomeration and therefore decrease in the crystallite size^51^. It is clearly noted that all the templated titania particles with grains of unequal sizes and uniformly distributed voids on the surface may bring out greater adsorption and hence better photo catalytic activity. Moreover uniformly distributed nano particles in the sample NT1 catalyst lead to better adsorption and hence good photo catalytic activity^52^.

**Figure 11:**
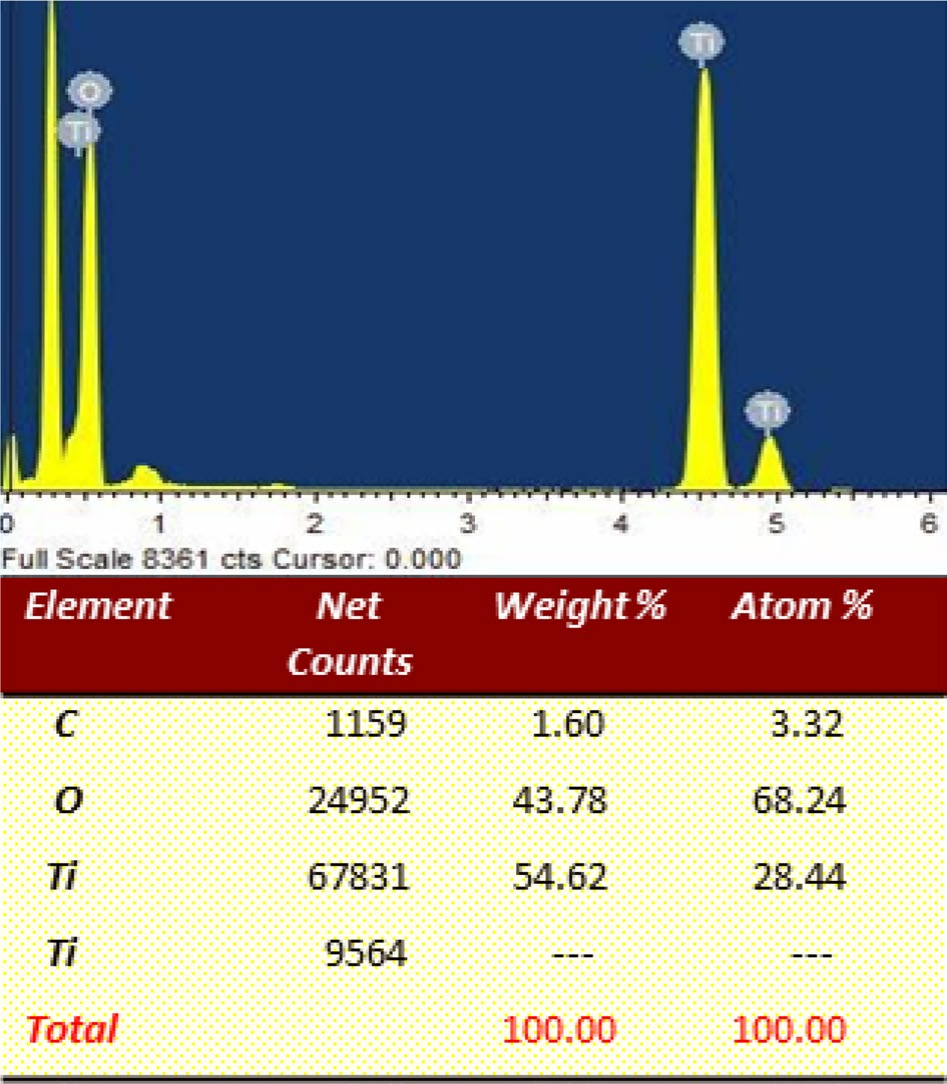
EDAX spectrum of NT1.

The elemental analysis of NT2 was inspected using EDAX spectrum as shown in Fig. 11. The EDAX spectrum clearly indicated the presence of peaks due to titania and oxygen^53^, which suggested the removal of organic residue like carbon from the template in the calcined NT1. The intense signal at 4.5 and 4.9 keV indicates that Ti is the major component of the sample^29^. The absence of peaks due to other elements clearly confirmed the purity of synthesized NT1 sample.

#### Transmission electron microscopy (TEM) analysis

**Figure 12:**
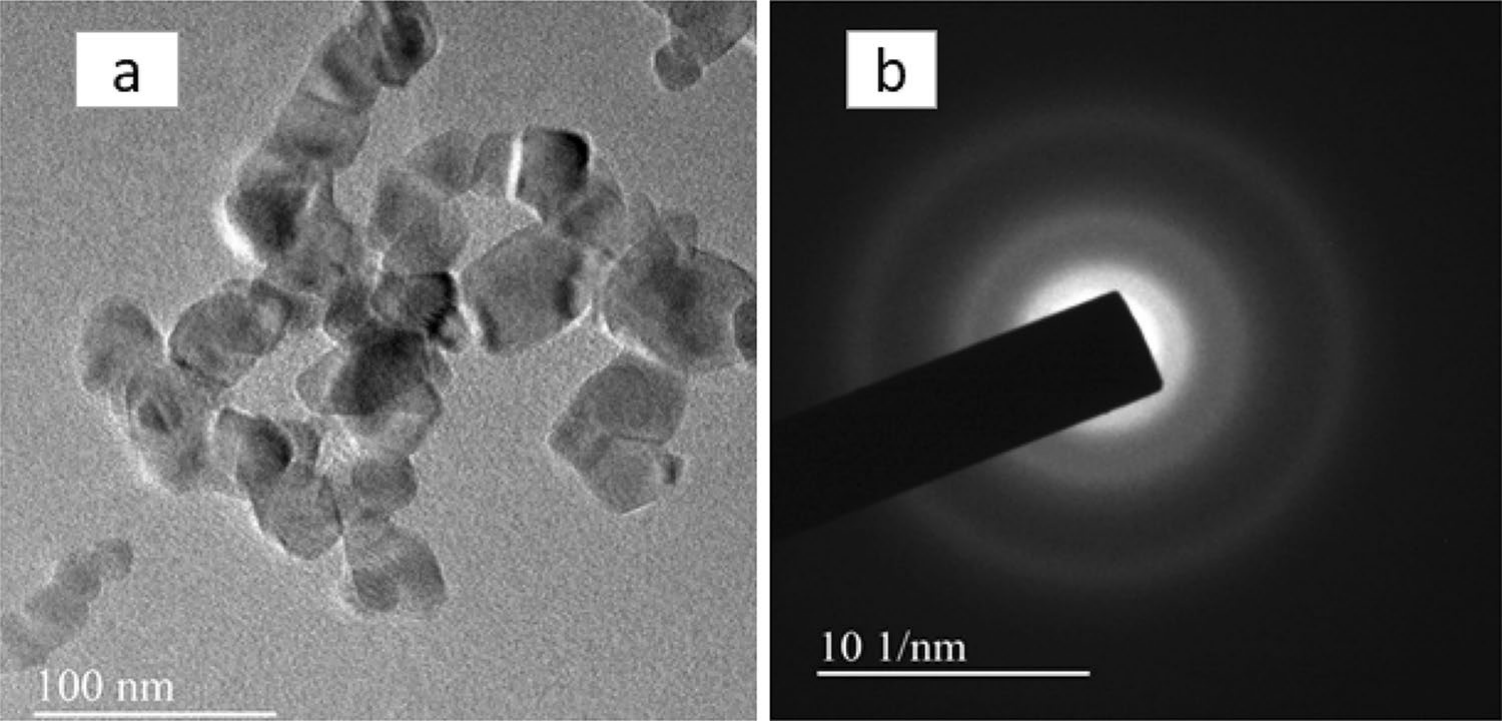
TEM image (**a**) and (b) SAED pattern of NT1.

**Figure 13:**
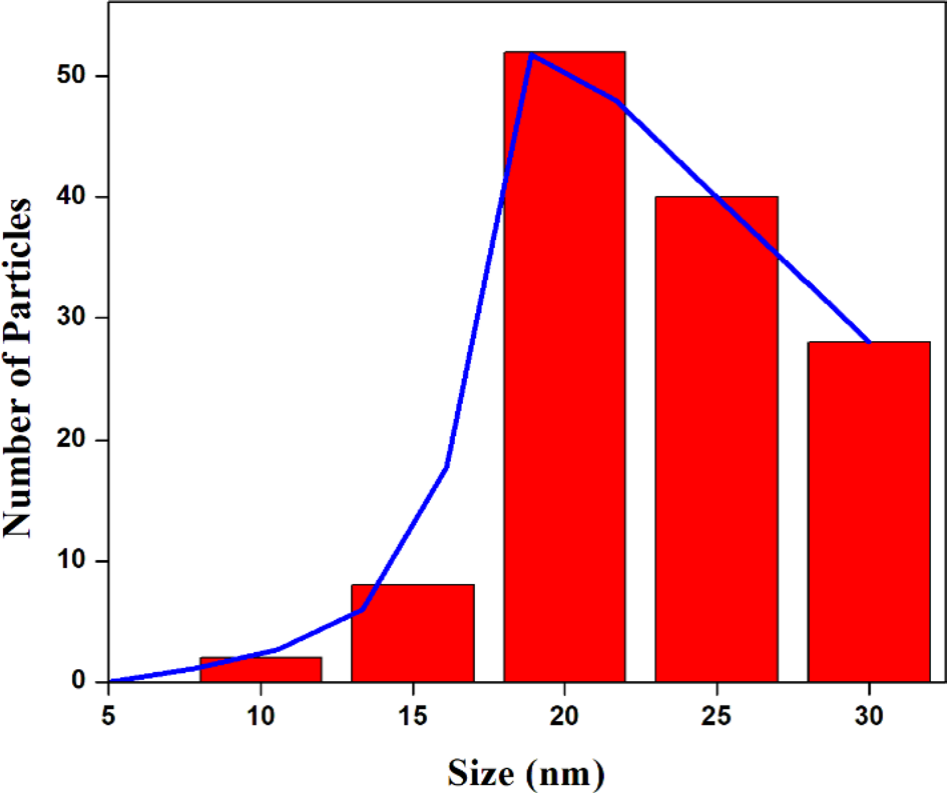
Particle size distribution histogram of NT1.

From the TEM image as shown in Fig. 12a, it is observed that the of templated titania particles calcined at 400 °C namely NT1 were with lowered agglomeration and indefinite shape in turn paved way for high stability in aqueous suspension^50^. Variation in the particle size was ranging about 18–28.8 nm, which was in good agreement with the XRD result analysis that as the calcination temperature increased crystallite size also increased reflecting on grain growth^54^. The particle size distribution of NT1 with respect to TEM analysis is shown in the Fig. 13. Highly resolved lattice plane fringes of anatase phase is shown in the Fig. 12b. SAED pattern of NT1 exhibited well defined ring patterns that were of typical anatase phase TiO_2,_ substantiated the purity as well as the crystallinity of the synthesized nano particles^55^. The highly crystalline grain with single spaced lattice rows were free from defects found to be coherent with X-ray diffraction analysis results^56^.

#### Photo catalytic studies

The photo catalytic efficiency of the synthesized sample was verified against Congo red, a textile dye, by irradiating 100 ml of dye solution containing specific quantity of templated NT samples under sunlight during 12 noon to 1 pm in the month of June. The intensity of sunlight was monitored using digital lux meter (HTC make Digital Lux meter, model LX-101A). The change in concentration of Congo red during the process of photo degradation was followed using UV–Vis Spectro photometer (Lambda 35, Perkin Elmer) at its absorbance maximum of 497 nm.

#### Photo catalytic efficiency of synthesized titania samples

**Figure 14:**
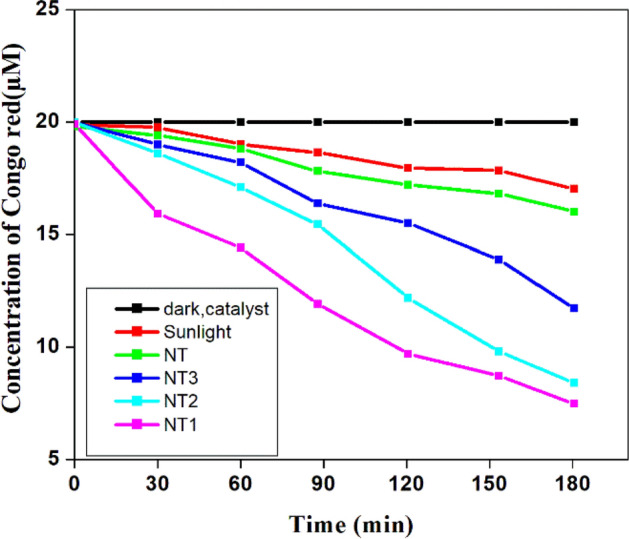
Degradation of Congo red with and without photo catalyst (Initial concentration = 20 µM, Catalyst dosage = 0.5 g l^−1^, pH = 4).

The efficiency of the photo catalysts thus derived are evaluated from the photo degradation of Congo red in the presence of Sun light and the results are shown in the Fig. 14. The photo degradation was effective only with the irradiated titania samples compared to direct photolysis and in the dark. It is shown that green calcined titania samples were 5–10% more effective than the samples without an organic template. The photo catalytic ability of the irradiated titania samples for the degradation of 20 µM Congo red was in the following order: NT1 > NT2 > NT3 > NT. It can be seen that NT1 calcined at 400 °C with the surface area of 98.6 m^2^ g^−1^ and an average crystallite size of 18 nm with moderate agglomeration displayed maximum photo catalytic efficiency (97.4) for the degradation Congo red. It is very interesting to note that the photo catalytic ability of NT2 (96.5%) is almost at par with NT1 inspite of low surface area and high crystallite size. This might be due to synergistic effect of anatase–rutile phases present in the sample, in extending the energy band gap and thereby increasing the retention of electron–hole pair life time and also producing more OH radicals on the surface^34^.

**Figure 15:**
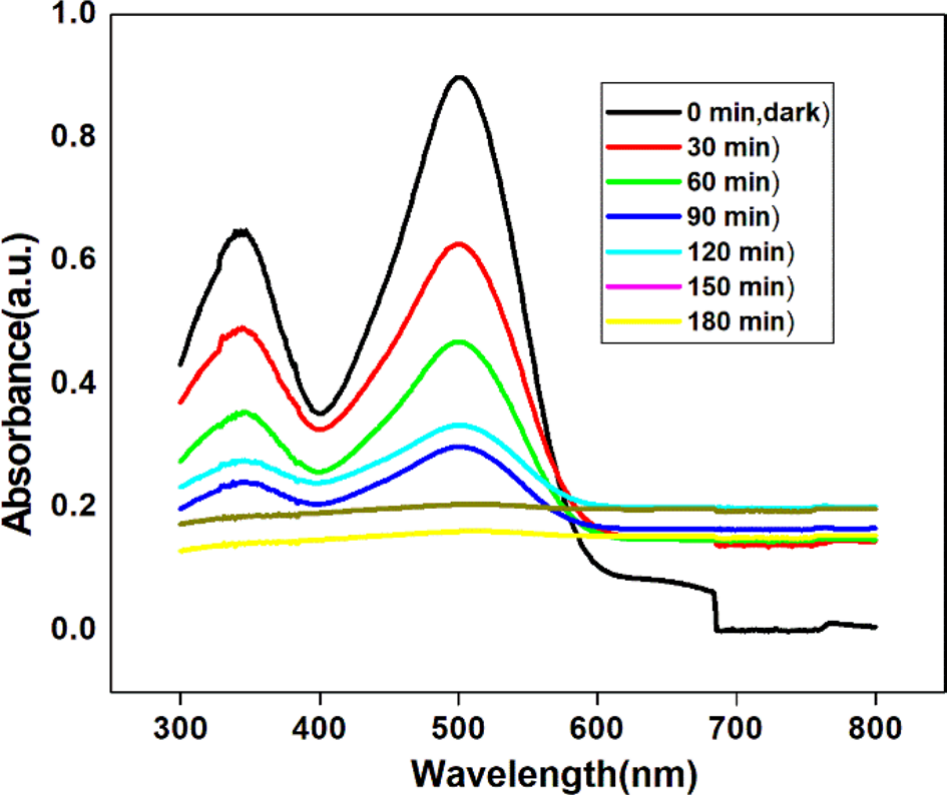
Absorbance spectrum of photo degradation of Congo red dye solution by NT1 (initial concentration = 20 µM, Catalyst dosage = 0.5 g l^−1^, pH = 4).

Figure 15, a UV–Vis spectra acquired from the photo degradation of Congo red using irradiated NT1 sample as a photo catalyst shows the decrease in the absorbance with the continuous removal of Congo red from the solution^57^. Thus elimination of chromophores of a pollutant in the visible region accompanied with the loss of benzene and naphthalene groups in the UV region has shown decrease in intensity and also the disappearance of bands with time^58^. The complete decolouration was noticed within 180 min due to partial degradation of pollutants and is indicated by a sharp intensed absorption edge^59^.

**Scheme 1:**
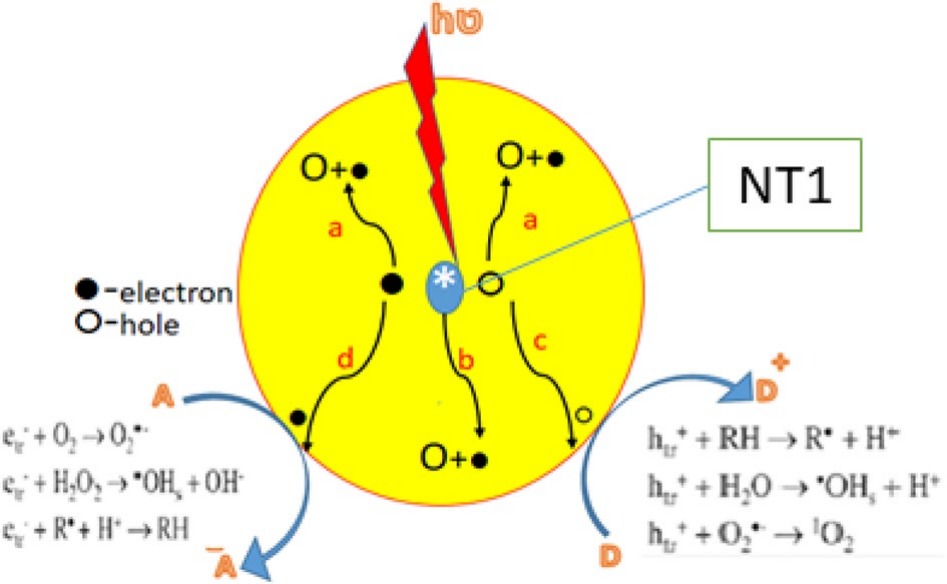
Mechanism of photocatalysis by NT1 catalyst under irradiation.

Scheme 1 explains the mechanism of photocatalysis by synthesized nano catalyst NT1 under solar irradiation. When a catalyst absorbs photon of energy equal to or greater than its band gap energy, pair of (h^+^, e^−^) generated thus move to the surface of a photocatalyst. This may either recombine or undergo redox reaction to degrade the pollutants in the aqueous medium. The electron and hole in the semiconductor surface react with the acceptor (step c) or donor (step d) respectively or may recombine at the trapped sites on the surface (steps a & b). Finally, the efficiency of a photocatalytic reaction is determined by the charge carriers, holes and electrons.

**Figure 16:**
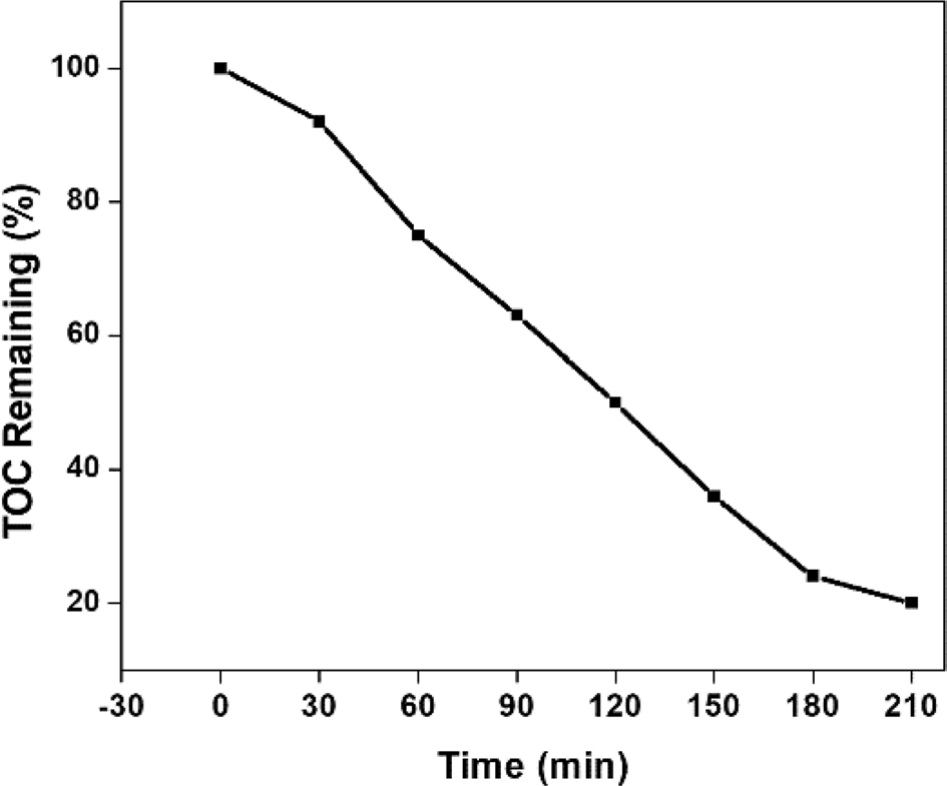
TOC removal efficiency of NT1 (initial concentration = 20 µM, Catalyst dosage = 0.5 g l^−1^, pH = 4).

Figure 16 shows whether the dye was only photo leached or photo degraded from the total organic content estimated using 20 µM Congo red solution and irradiated NT1. The 100% decolouration and about 80% reduction in the carbon content by 180 min was noted from the solar light induced process. The graphical abstract clearly explain the tentative mechanism of Congo red degradation in visible light region. On irradiation of NT1, valence band electron gets excited and moved to conduction band resulting on electron–hole pair. These excitons are responsible for the generation of active species like OH, ·O^2–^, which in turn decompose Congo red into CO_2_, H_2_O and NO_3_^–^ etc.^43^. This showed the partial degradation of dye to a profitable extent using a catalyst.

#### Effect of calcination temperature

**Figure 17:**
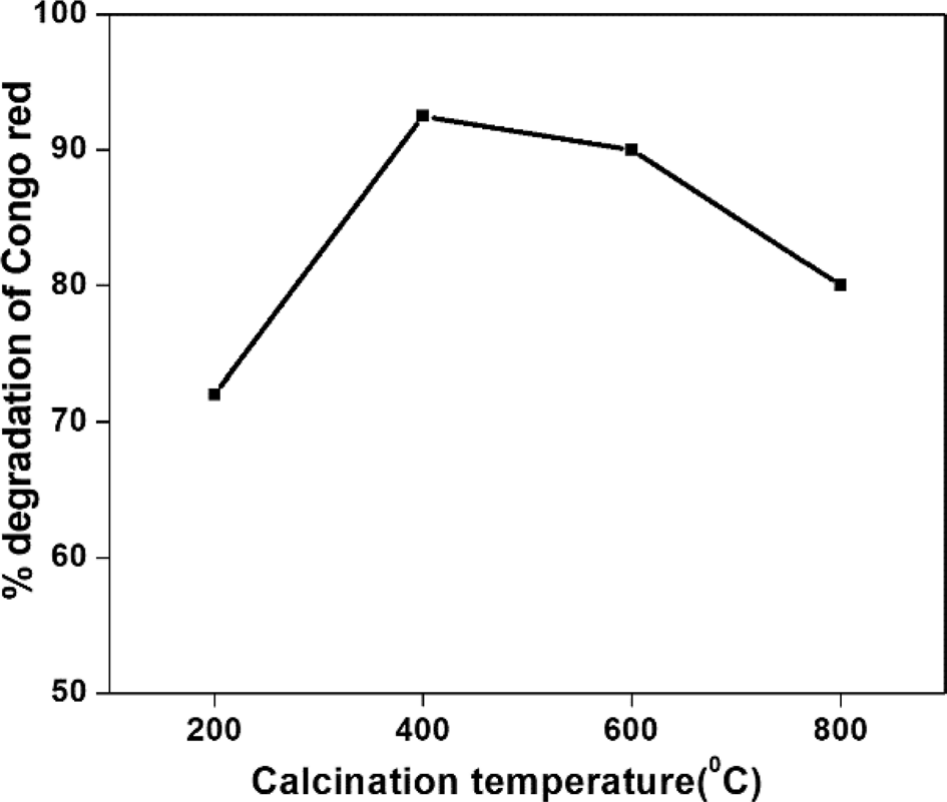
Effect of calcination temperature of NT1 on the photo degradation of Congo red [Congo red] = 20 µM, pH = 4, Amount of NT1 = 0.5 g l^−1^.

The photo degradation ability of NT1 catalyst calcined at 200, 400, 600 and 800 °C is shown in Fig. 17. Generally, calcination at a higher temperature brings out dehydration of TTIP (TiO_4_H_8_ → TiO_2_ + H_2_O) creating more active sites on the surface, therefore initiate more adsorption of pollutants and hence degradation of a pollutant^60^. The photo catalytic degradation ability of the calcined sample with respect to Congo red showed an increase with the increase in calcination temperature from 200 to 400 °C, and then decreased beyond this. Congo red was degraded to about 92.5, 90 and 80.5% under 180 min of solar irradiation in presence of NT1 calcined at 400, 600 and 800 °C, respectively. At 800 °C, the decreased photo degradation of Congo red observed might be due to the nearing completion of anatase phase to rutile phase transformation^60^ and at 200 °C because of organic residual impurities remain undecomposed on the surface. Hence, a calcination temperature of 400 °C was found to be optimal for the analysis using a templated titania.

#### Effect of catalyst dosage

**Figure 18:**
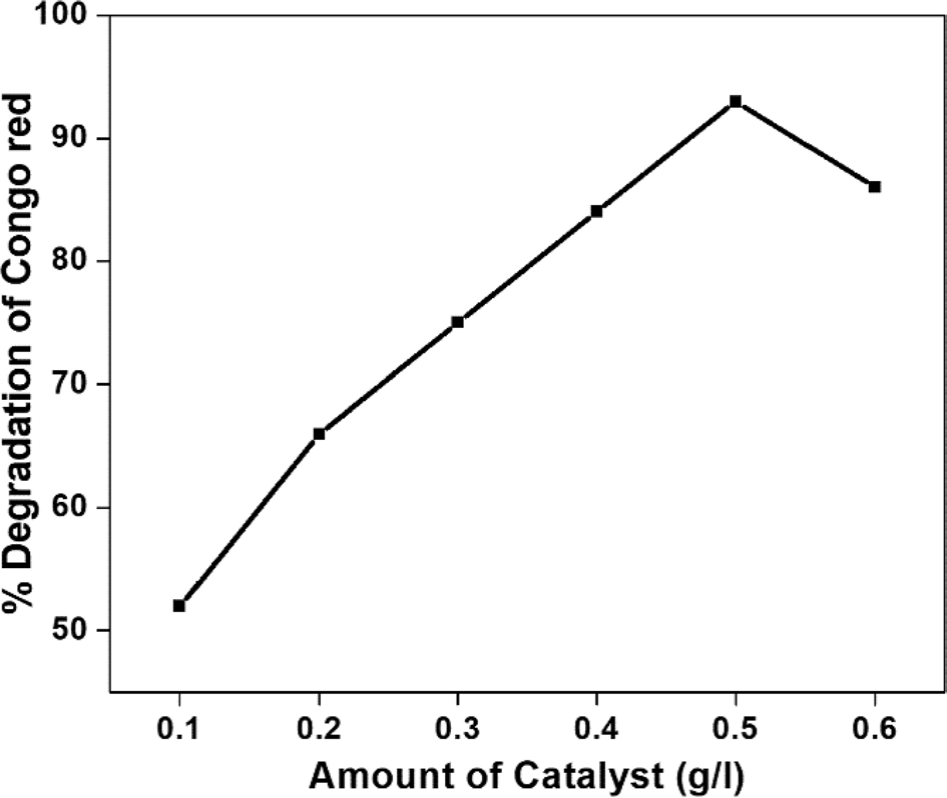
Effect of Catalyst Dosage on the photo degradation of Congo red [Congo red] = 20 µM, pH = 4, Amount of NT1 = 0.5 g l^−1^.

The effect of catalyst NT1 loaded for the photo degradation of Congo red is shown in the Fig. 18. The degradation percentage got increased from 51.5 to 92.8%, when the amount of catalyst was increased from 0.1 to 0.5 g l^−1^, resulted in more active sites available on the catalyst surface which in turn enhanced the absorption of light for better photo degradation^61^. There was a decrease in the rate of degradation of the dye when the catalyst dose was increased further beyond 0.5 g l^−1^, due to aggregation of catalyst particles that would block the pores of a catalyst and hence the extent of penetration of light^62^. Hence, 0.5 g l^−1^ loading of NT1 was found to be optimum under current experimental conditions for the photo catalytic degradation of Congo red.

#### Effect of pH

**Figure 19:**
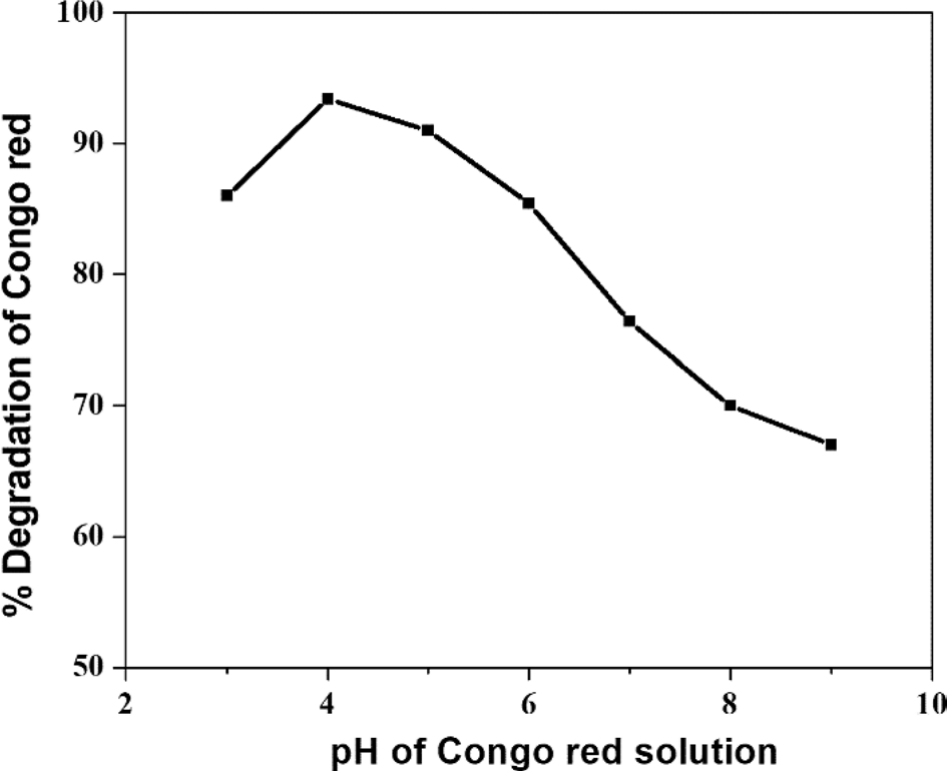
Effect of pH on the photo degradation of Congo red [Congo red] = 20 µM, Amount of NT1 = 0.5 g l^−1^.

Figure 19 shows the effect of pH on the rate of photo degradation under varying range of the pH of the solution from 3 to 10 and other experimental factors were kept constant. The degradation of aqueous Congo red solution increased from 85.2 to about 93.4% when pH of the solution was increased from 3 to 5 and the same got decreased to 69.1% at pH = 10. The zero point charge of titania is around 6.8 and hence its surface remain to be acidic, positively charged due to protonation below this pH. It would become basic, negatively charged because of deprotonation, above this pH^63^. During photodegradation in acidic medium (low pH) the anionic Congo red, as a strong Lewis base got adsorbed easily on the positive titania surface^64^. But in alkaline medium, the extent of adsorption got reduced due to negative surface charge of titania and thus resulted in lessened degradation^63^.

#### Effect of initial dye concentration on the degradation of Congo red and kinetic studies

**Figure 20:**
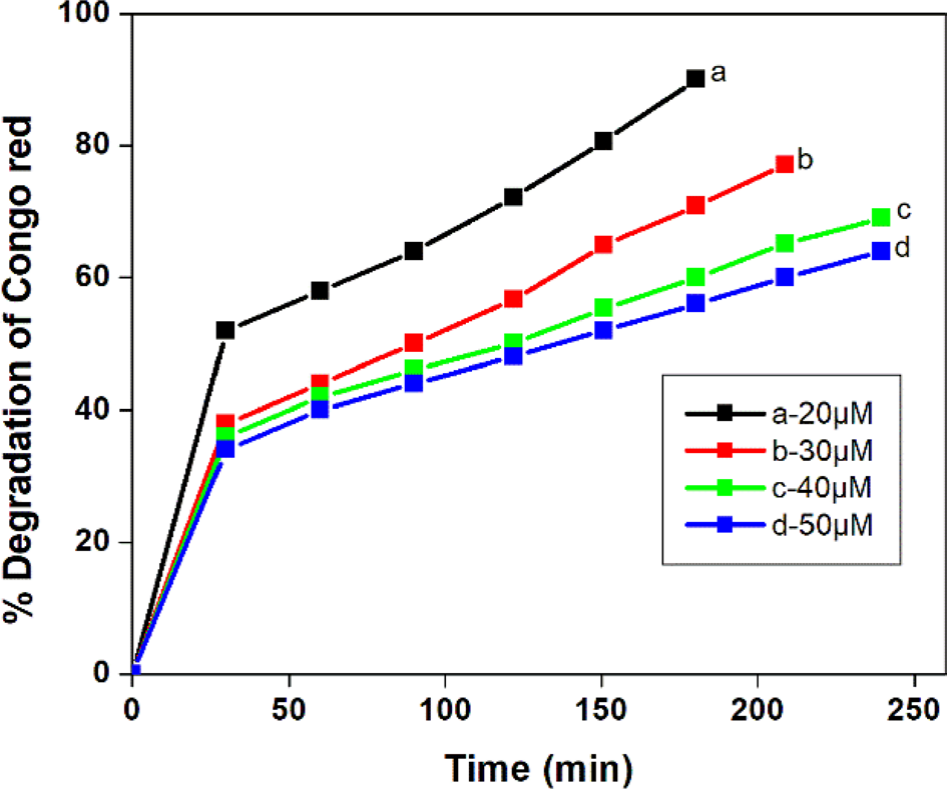
Effect of Initial dye concentration on the photo degradation of Congo red dye solution (pH = 4, NT1 = 0.5 g l^−1^).

Figure 20 shows that when concentration the dye increased from 20 to 50 µM, the initial rate of photo catalytic degradation decreased. This is because as the initial dye concentration increased, saturation of the active sites due to more availability of pollutants on the surface of titania takes place. Also the presence of more number of dye molecules in the bulk solution might have absorbed the radiation, and therefore less number of OH radicals were made available on the surface of the catalyst and subsequently the degradation percentage of the organic substrate was reduced^65^.

**Figure 21:**
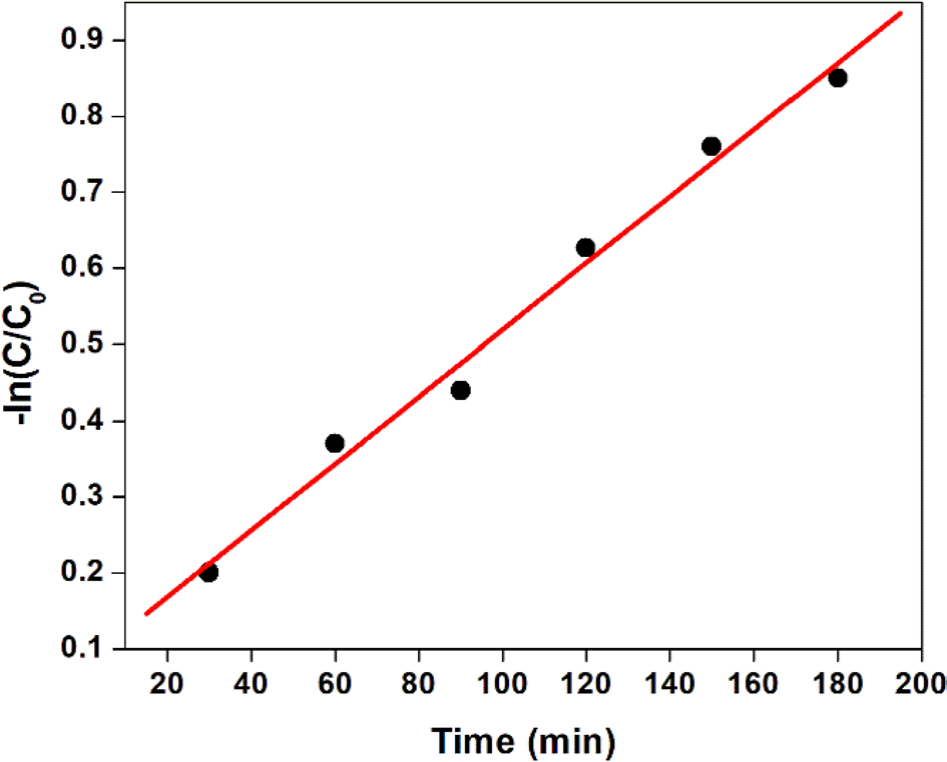
Kinetic study of photo degradation of Congo red using NT1.

Figure 21 represents the pseudo-first order kinetics of photo catalytic degradation of Congo red with respect to the dye concentration. In a plot of − ln (C/C_○_) versus t, a linear relation between the dye concentration and irradiation time has been observed where C_○_ is the equilibrium concentration of the dye and C is the concentration at time t. The first order rate constant was estimated as 0.0154 min^−1^ using the rate expression − ln [C/C_○_] = k′t^63^.

**Figure 22:**
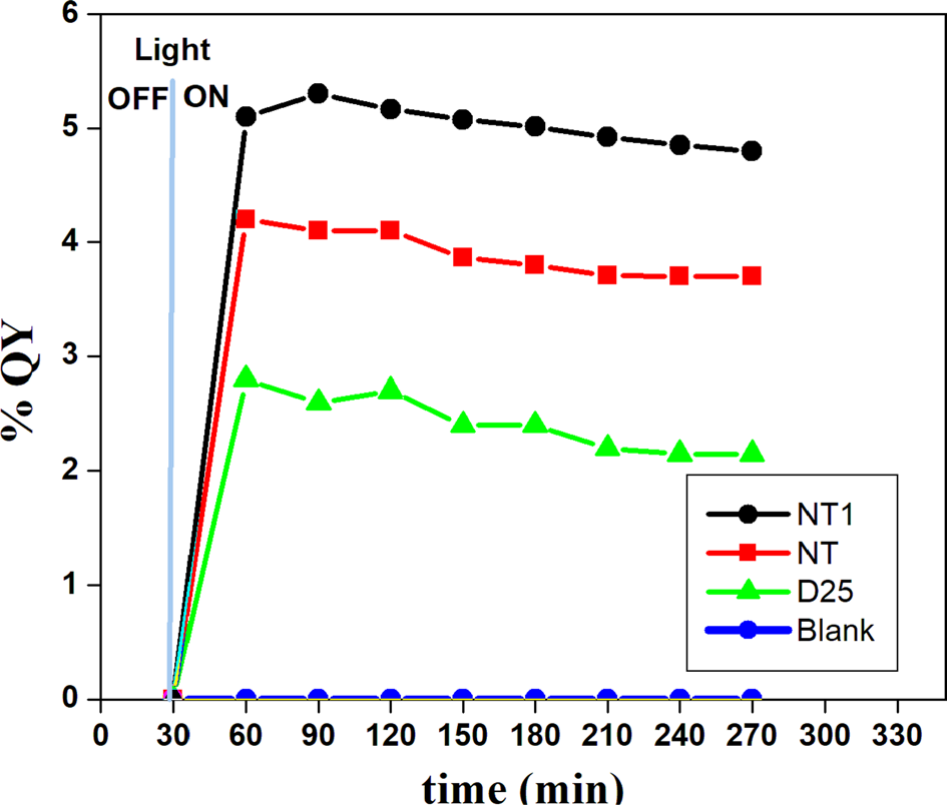
Overall QY of the samples at pH = 4 using Argon as purging gas.

Figure 22 gives the quantum yield percentage of NT and NT1 samples, Degussa-25 and blank solution with respect to hydrogen ion formation. It is interesting to note the progressive increase in the H_2_ ion production and therefore the stability of quantum yield throughout the cycle. It is observed that at pH = 4, due to protonation no decrease in the rate of hydrogen ion supply to the catalyst’s surface and hence better photocatalytic effect in the acidic medium^66^. Of all synthesized samples NT1 has shown maximum quantum yield of 5.3.

#### Reusability of NT1

**Figure 23:**
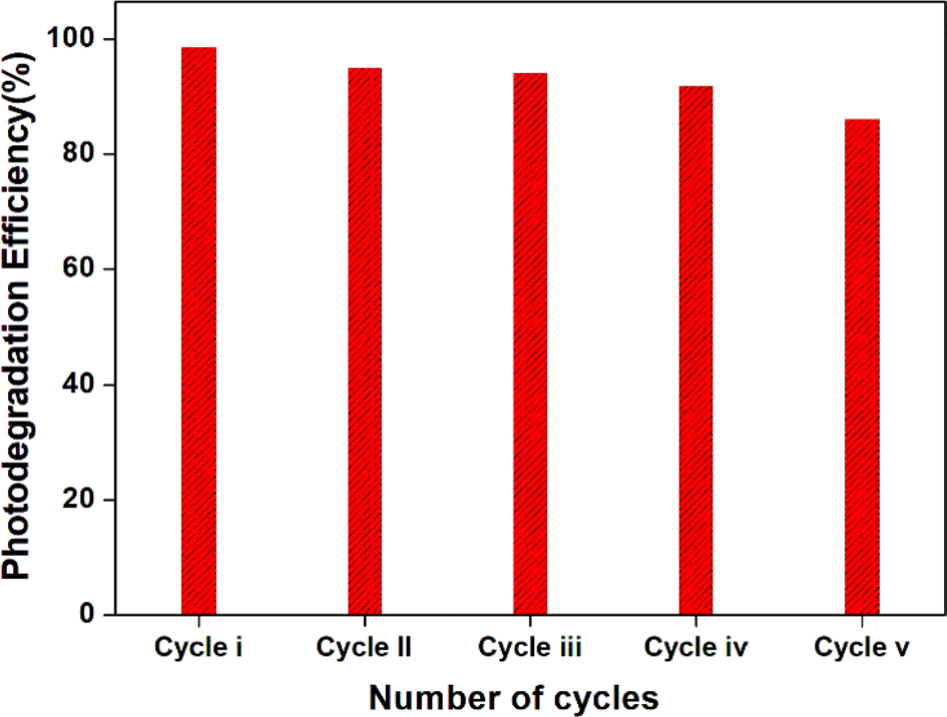
Recycling of photo degradation of Congo red solution using NT1.

From Fig. 23, the reusability of NT1 was examined to study the efficacy and stability of the synthesized nano titania catalyst. A catalyst was collected, filtered, also washed repeatedly with distilled water and dried at 60–70 °C in an oven, at the end of photo catalytic process. The reclaimed catalyst was employed minimum for five cycles for the photo degradation of Congo red repeatedly. Only a negligible reduction in the photo catalytic efficiency was observed up to four times of recycling. This verified that during the repeated recycling and reuse of synthesized photo catalyst it was found to be stable, efficient, and relatively unaffected. Marginal decrease in the activity of the regained synthesized samples during repeated recycling might be due to the removal of active species from the catalyst’s surface during washing and recovery processes^67^.

## Conclusion

The influence of calcination temperature over the formation, properties and photo catalytic activity of leaf extract templated nano titania samples by sol–gel technique was explored clearly in this work. The profound influence of calcination along with an organic template is evidenced from the optical, crystallite surface and morphological properties of synthesized titania samples. Among the titania samples prepared NT1 (calcined at 400 °C), of only anatase phase, with maximum surface area (98.6 m^2^ g^−1^) and minimum crystallite size (18 nm) has exhibited 97.7% photo degradation of Congored in the visible light region due to bathochromic shift resulted in. The sample NT2 (calcined at 600 °C) of both anatase and rutile phases also displayed excellent photo degradation about 96.5% against Congored under Sun light, clearly indicated the synergistic effect of mixed phases in extending the absorption edge to the visible light region. Thus to summarize, the observed experimental results of the present work highlighted the significant impact of calcination in tuning photo catalytic ability of bio-templated titania samples which can be further explored in the field of sensing, drug delivery, self-cleaning and anti-fouling coatings in future apart from photo catalytic applications.
